# Secondary Nucleation of Aβ Revealed by Single‐Molecule and Computational Approaches

**DOI:** 10.1002/advs.202404916

**Published:** 2024-08-19

**Authors:** Nathan Meyer, Nicolas Arroyo, Lois Roustan, Jean‐Marc Janot, Saly Charles‐Achille, Joan Torrent, Fabien Picaud, Sébastien Balme

**Affiliations:** ^1^ Institut Européen des Membranes UMR5635 University of Montpellier ENCSM CNRS Place Eugène Bataillon Montpellier 34095 France; ^2^ UR SINERGIES University of Besançon 16 route de Gray Besançon 25000 France; ^3^ INM University of Montpellier INSERM Montpellier 34090 France

**Keywords:** amyloid, confocal fluorescence spectroscopy, nanopore, secondary nucleation, single molecule

## Abstract

Understanding the mechanisms underlying amyloid‐β (Aβ) aggregation is pivotal in the context of Alzheimer's disease. This study aims to elucidate the secondary nucleation process of Aβ42 peptides by combining experimental and computational methods. Using a newly developed nanopipette‐based amyloid seeding and translocation assay, confocal fluorescence spectroscopy, and molecular dynamics simulations, the influence of the seed properties on Aβ aggregation is investigated. Both fragmented and unfragmented seeds played distinct roles in the formation of oligomers, with fragmented seeds facilitating the formation of larger aggregates early in the incubation phase. The results show that secondary nucleation leads to the formation of oligomers of various sizes and structures as well as larger fibrils structured in β‐sheets. From these findings a mechanism of secondary nucleation involving two types of aggregate populations, one released and one growing on the mother fiber is proposed.

## Graphical abstract

Experimental single‐molecule techniques and computational methods are used to investigate the secondary nucleation of amyloi‐d‐β. By investigating the different oligomer species and the effect of seeds, a mechanism of secondary nucleation involving two types of aggregate populations, one released and one growing on the mother fiber, is proposed.

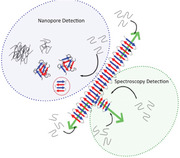

## 1 Introduction

Alzheimer's disease (AD) is a neurodegenerative disorder that is characterized by the accumulation of amyloid plaques in the brain. The self‐aggregation of amyloid‐β (Aβ) peptide into oligomers and amyloid fibrils is usually divided into three phases: the lag and growth phases before reaching a plateau.^[^ ^1^, ^2^ ^]^ All these phases involve multiple parallel processes (i.e., primary nucleation, elongation, secondary nucleation, and fragmentation) that occur at different rates,^[^ ^3^ ^]^ during which oligomers can adopt a wide range of transient structures before their self‐organization into fibrils rich in β‐sheet structures.^[^ ^4^ ^]^ Secondary nucleation involves the formation of new nuclei or fibrils from the existing ones.^[^ ^5^ ^]^ This contributes to an increase in the nucleus proliferation during the lag phase and bypasses the primary nucleation process.^[^ ^3^ ^]^ This leads to the multiplication of short‐lived oligomeric intermediates that cause neurotoxicity^[^ ^6^ ^]^ and proliferation of fibrils in the brains of AD patients.^[^ ^7^ ^]^ Therefore, understanding secondary nucleation has attracted increasing attention in recent years.

Secondary nucleation is dependent on several factors, such as pH, salt, and inhibitor.^[^ ^8^ ^]^ The properties of preformed aggregates (called seeds) influence the kinetics, structure, and polymorphism of newly formed Aβ fibrils.^[^ ^7^ ^]^ Indeed, by acting as a template,^[^ ^9^ ^]^ the seeds lead to the formation of new fibrils with distinct thermodynamic signatures.^[^ ^10^ ^]^ The catalytic surface of the seed may be located along the sides of the fibrils rather than at their ends.^[^ ^10^ ^]^ It should be efficient only between peptides of identical morphology to explain the structural conversion at the fibril surface.^[^ ^11^ ^]^ This was supported by a study using molecular dynamics simulations, which showed that the Aβ42 monomer undergoes structural changes when adsorbed onto the fibril surface.^[^ ^12^ ^]^ Another study suggested that the accumulation of amyloid fibrils is coupled to the generation of low‐molecular‐weight diffusive aggregates from monomeric peptides.^[^ ^13^ ^]^ Recently, high‐resolution microscopy revealed that monomers grow into relatively large aggregates on fibril surfaces along the length of fibrils and, at the same time, small oligomers with incompatible structures are released into the solution.^[^ ^6^ ^]^

Numerous techniques have been used to investigate amyloid growth. The fluorescence of Thioflavin T (ThT) can be processed in bulk or at the single‐molecule level using confocal fluorescence spectroscopy.^[^ ^14^, ^15^ ^]^ A limitation of ThT is its specificity for probing β‐sheet structures. Transmission electron microscopy (TEM) and atomic force microscopy (AFM) provide high‐resolution structures of amyloids,^[^ ^9^ ^]^ whereas small‐angle X‐ray scattering (SAXS) is suitable for structural investigation of fibril elongation.^[^ ^16^ ^]^ Tip‐enhanced Raman spectroscopy (TERS) allows distinction between parallel and antiparallel β‐sheets.^[^ ^17^ ^]^ Among alternative techniques, solid‐state nanopores are one of the most interesting, as shown by the growing interest in this technology in recent years.^[^ ^18^, ^19^ ^]^ The detection of a protein or aggregate translocating through a single nano‐aperture by recording the electrical perturbation allows the estimation of the volume.^[^ ^20^, ^21^ ^]^ Various types of artificial nanopores have been used to investigate amyloids. SiN nanopores with a low aspect ratio allow the direct measurement of oligomers in solution.^[^ ^22^, ^23^ ^]^ Monitoring the frequency and magnitude of current perturbations can provide information about the kinetics of oligomer aggregation^[^ ^24^ ^]^ and polymorphism.^[^ ^25^ ^]^ Nanopores with high aspect ratios (nanopipettes and track‐etched polymers) are interesting alternatives to SiN nanopores because of their robustness and low cost.^[^ ^26^ ^]^ Using such nanopores, it was shown that the length of the amyloid fibrils affects the translocation dynamics, and thus, the distribution of the relative current blockade amplitude.^[^ ^27^, ^28^, ^29^, ^30^ ^]^ Owing to their geometry, their resolution is lower than that of SiN; however, by using a set of nanopipettes with different diameters and suitable geometrical models, it is possible to probe oligomers with a wide range of volumes.^[^ ^31^ ^]^ More recently, our group introduced a new concept called real‐time fast amyloid seeding and translocation (RT‐FAST) to detect the presence of preformed aggregates in a solution by direct incubation with monomers inside the reservoir of a nanopipette.^[^ ^32^, ^33^ ^]^ The interesting feature of this method is that the oligomer was detected during the lag phase, regardless of its structure. Based on this, we hypothesized that RT‐FAST allows for the analysis of the small oligomer volume generated by secondary nucleation at the early stage of aggregation. By combining this information with confocal fluorescence spectroscopy, it is possible to draw a map of the species generated by secondary nucleation of Aβ.

**Figure 1:**
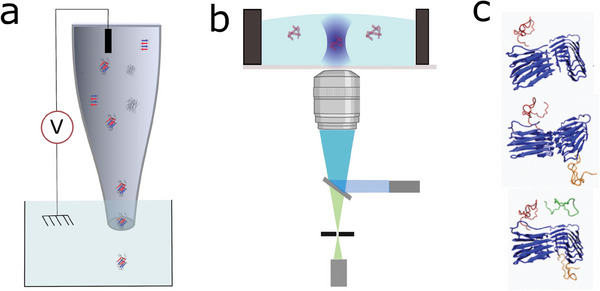
a) Sketch of nanopipette experiments. b) Sketch of the experimental confocal fluorescence spectroscopy. c) Simulated MD systems of fibril 5OQV (blue) with 1, 2, or 3 monomers (red, orange, and green) placed around the fibril. Closest atoms between fibril and respectively the red, orange, and green monomers are distanced of ≈4, ≈6, and ≈10 Å.

This study aims to further understand amyloid‐β seeding and propose a mechanism for secondary nucleation. We were interested in the lag phase, which is less known because most of the species present are not sensitive to ThT. To this end, we first characterized the kinetics of Aβ42 peptides aggregation when preformed seeds collected at different stages of maturation (growing and plateau phases, unfragmented and fragmented) were added, using classical bulk techniques involving ThT fluorescence. We then used two single‐molecule techniques to characterize oligomer distribution over time (**Figure** **1**). The first involved the use of nanopipettes with different diameters to detect all species within a range of diameters, regardless of their structures. The second technique involves confocal fluorescence spectroscopy to probe oligomers with a β‐sheet structure. Then, the experimental results are compared to molecular dynamic (M simulations and finally discussed to propose a secondary nucleation mechanism.

## 2 Results and Discussion

### 2.1 Seeding Capacity of Fibers Studied by Bulk Method

**Figure 2:**
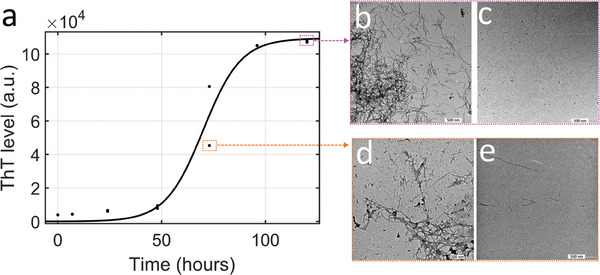
a) Aggregation kinetics of Aβ42 peptides. Transmission electron microscopy image of samples collected after 120 h b) before and c) after sonication. TEM image of samples collected after 73 h of incubation d) before and e) after sonication.

One purpose was to compare how the nature of the preformed fibers influences the seeding capacity and thus influences the secondary nucleation process. To do this, the production of preformed aggregates was carried out by incubating Aβ42 monomers at 37 °C at two incubation times of 73 and 120 h, corresponding to the growth and plateau phases respectively (**Figure** **2**a). To determine whether the secondary nucleation process was limited by seed diffusion or reaction at the fiber surface, the preformed aggregates were fragmented by sonication. Indeed, assuming that secondary nucleation is a process limited by the reaction at the lateral surface of fibers and not by the diffusion of aggregates, fragmentation should have no effect on the species detected during the aggregation process. TEM images of the samples collected after 73 h showed amyloid fibrils and clusters that were still forming. After fragmentation for 1 h at 34 W, only fibers shorter than 200 nm were observed. The amyloid fibers were mature and formed clusters in the sample collected after 120 h of incubation. After sonication for 20 min at 100 W, the TEM images revealed that the amyloid fibers were fragmented into fibrils of approximately 30 nm (Figure 2b–e).

**Figure 3:**
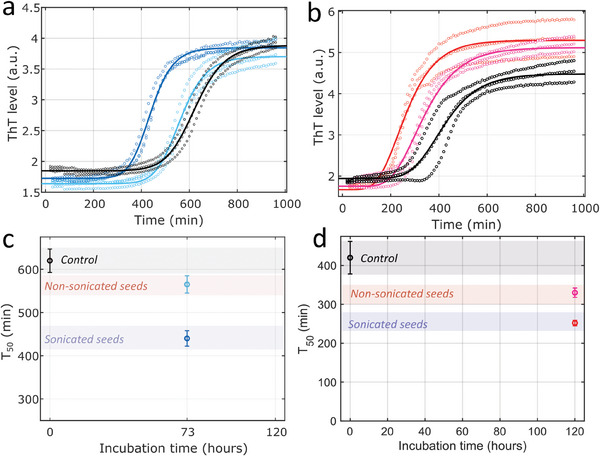
a) Aggregation kinetics of Aβ42 peptides in microplates in the absence (black) and in the presence of seeds harvested a) in growing phase (after 73 h of incubation) before (cyan) or after 1 h of fragmentation at 34 W (blue) and b) in plateau phase (after 120 h of incubation) before (pink) or after 20 min of fragmentation at 100 W at 4 °C (red). T_50_ of the different kinetics obtained by fitting kinetic using Boltzmann equation for sample in presence of seed harvested in c) growth phase and d) plateau phase.

To study the seeding capacity of the preformed aggregates, the kinetics of Aβ42 in a microplate were followed by ThT fluorescence emission measurement (*I_t_*) as a function of incubation time (**Figure** **3**). The experimental data were fitted using the Boltzmann equation to determine T_50_ (Equation (1)):

(1) $$I_{t}=\frac{I_{i}-I_{f}}{1+e^{\left(t-T_{50}\right)}}\;+I_{f}$$

where *I_i_* and *I_f_* are the fluorescence intensities at the initial and plateau levels, respectively. For the control condition (no seeds added), the T_50_ was reached after 630 ± 20 min of incubation. The addition of 1% seed incubated for 73 h reduced the T_50_ to 570 ± 10 min. Seed fragmentation further reduced T_50_ to 427 ± 15 min (Figure 3a,c). These results were confirmed by experiments in which seeds harvested after 120 h of incubation were used (Figure 3b,d). In this set of experiments, the T_50_ of the control condition was 420 ± 45 min. For the conditions incubated in the presence of 1% unfragmented and fragmented seeds, the T_50_ was reached after 332 ± 7 and 240 ± 3 min, respectively. There was a noticeable difference between the T_50_ values for the two control conditions. This is not surprising because different batches of Aβ peptides can behave differently with variations in the aggregation time.^[^ ^34^ ^]^ However, our findings confirmed that the addition of preformed aggregates reduced aggregation time. Furthermore, it was observed that seeds harvested in the growing phase (73 h) exhibited a lower catalytic aggregation efficiency than mature fibers (120 h), with a T_50_ decrease of 9% compared with 21%. This variation was also noted for fragmented seeds, where the T_50_ decreased by 32% as opposed to 42% for seeds harvested after 73 and 120 h of incubation. These findings are in line with those of several previous studies showing that mature amyloid fibers are more efficient in accelerating the aggregation process than oligomers.^[^ ^35^, ^36^ ^]^ Measuring bulk ThT fluorescence allows access only to the aggregation kinetics of amyloid‐structured β‐sheets. Thus, the reduction of the lag time can involve two mechanisms. Seed fragmentation leads to an increase in the number of ends where the monomers can assemble in a β‐sheet arrangement. On the other end, the secondary nucleation assumes a catalytic role of the seed surface to produce new oligomers. The bulk measurement of ThT fluorescence does not provide information on the size distribution of oligomers, regardless of their structure, which is mandatory for further understanding of the secondary nucleation mechanism. Access to this information requires the use of a single‐molecule technique to differentiate the end‐on (structured β‐sheet) and end‐off or amorphous aggregates formed during the lag phase.

### 2.2 Analysis of Aβ42 Oligomers Formed During the Lag Phase Using Nanopipette

**Figure 4:**
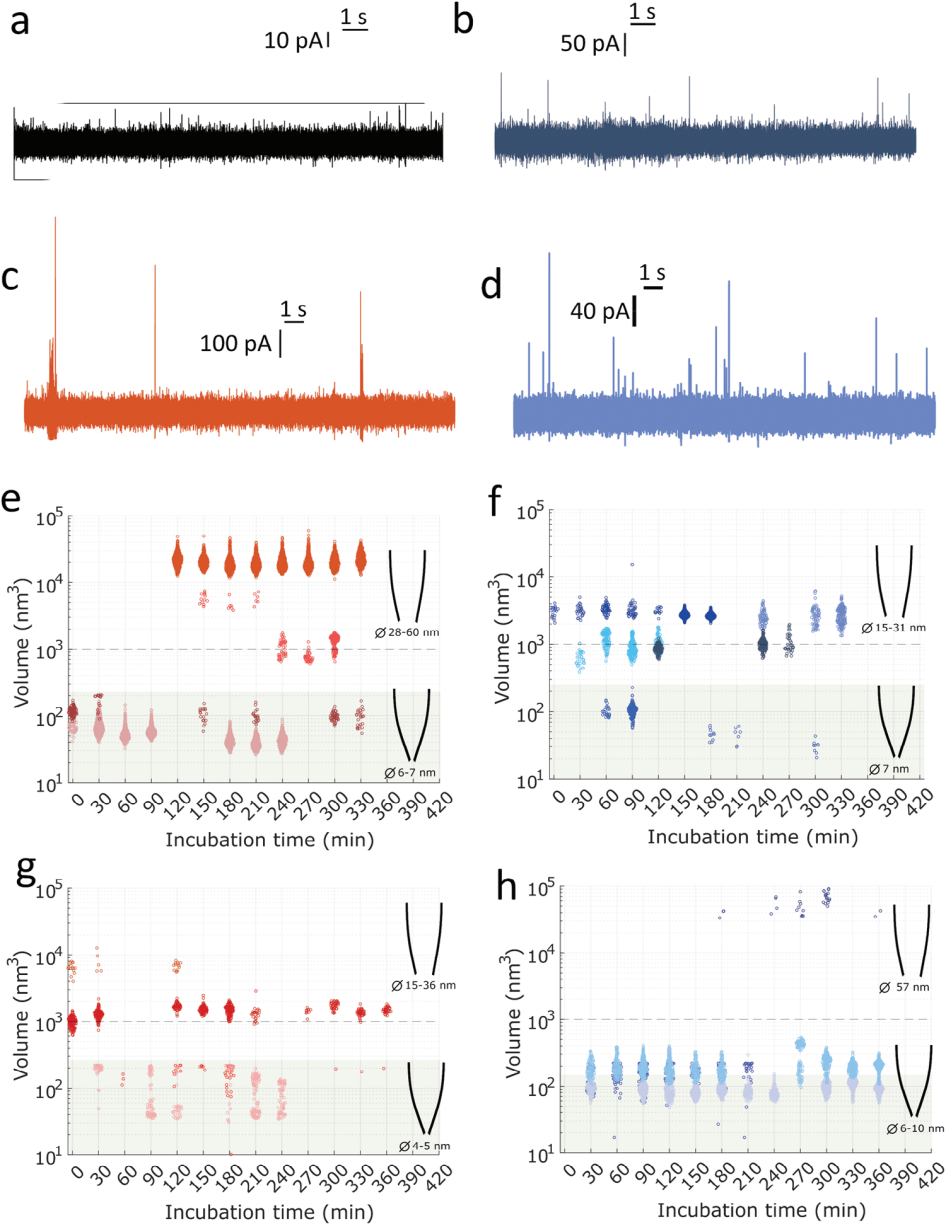
Example of current traces recorded after 180 min incubation in the pipette for the condition seeded with 1% seeds harvested in growing phase (after 73 h of incubation) a) unfragmented and b) fragmented and seeds harvested in plateau phase (after 120 h of incubation) c) unfragmented and d) fragmented. Volume of amyloid species detected as a function of incubation time for the condition seeded with 1% seeds harvested in growing phase (after 73 h of incubation) e) unfragmented and f) fragmented and seeds harvested in plateau phase (after 120 h of incubation) g) unfragmented and h) fragmented.

Oligomers formed during the lag phase were characterized independently of their structure (unlike ThT labeling) in real‐time using nanopipettes. Therefore, the RT‐FAST method was used in this study. It involves incubating Aβ42 monomers with or without seeds (reproducing the experimental conditions used for microplate experiments) directly in the reservoir of the nanopipette. After 30 min, a constant voltage of −500 mV was applied by an electrode inserted into the pipette to drive Aβ42 oligomers, which were negatively charged at pH 7.2, by electrophoretic flow. For each condition, a set of nanopipettes with different diameters was used to characterize the complex mixture because it was demonstrated that only oligomers with diameters ranging between the radius and diameter of the nanopipette could be detected^[^ ^31^ ^]^ (Figure S1 and Table S1, Supporting Information). The volume of each detected oligomer was deduced from the blockage‐level value (ΔI/I_0_) and nanopipette properties (radius *r_p_* and angle α, determined for each nanopipette) using a previously reported^[^ ^31^ ^]^ geometric model (Equations (2) and (3)). One assumes cylindrical geometry of the oligomer with length (*L_o_*) and radius (*r_o_*) for those *L_o_* =  2*r_o_*

(2) $$\frac{\Delta I_{\max}}{I}=\frac{G^{-1}}{G^{-1}-R_{\max}}\;$$

(3) $$\begin{array}{ccc} R_{\max} & = & \;\frac{1}{\left(\kappa \pi r_{p}\left(r_{p}+2r_{0}a\right)/2r_{0}\right)-\left(1-\left({r_{0}}^{2}/2r_{p}\left(r_{p}+2r_{0}a\right)\right)\right)}\\ & & -\frac{1}{G}-\frac{2x}{\kappa \pi r_{p}\left(r_{p}+\left(2r_{0}\right)a\right)} \end{array}$$

where G is the conductance of the nanopore without aggregates. By solving Equations (2) and (3) for each blocking event, we obtained the volume distributions for all experimental conditions plotted in **Figure** **4**.

For the control, the current traces did not exhibit blockade during 360 min of Aβ42 monomer incubation, regardless of the nanopipette diameter, ranging from 10 to 30 nm. This is consistent with our previous findings^[^ ^32^ ^]^ and can be explained by the formation of aggregates that are either too small or not sufficiently concentrated to be detected. As no event was detected with nanopipettes with a diameter of 10 nm, we can assume that the concentration of aggregates was too low to induce events during the acquisition time (10 min). For Aβ42 aggregation experiments containing 1% unfragmented seeds from the growth phase (incubated for 73 h), current blockades were observed from the first 10 min of incubation for nanopipettes with diameters smaller than 10 nm. Using larger nanopipettes (diameters of approximately 30 ± 3 nm), events appeared after 120 min of incubation. The volume of the aggregates characterized by small nanopipettes (*d_p_* < 10 nm) was smaller than 200 nm^3^ corresponding to an oligomer smaller than 6 nm. The use of larger nanopipettes (*d_p_* > 25 nm) revealed a dispersed distribution of oligomers with volumes ranging from 500 to 50 000 nm^3^ corresponding to oligomer sizes between 8 and 40 nm. The first series of experiments showed that small oligomers were detected before those with larger diameters (Figure 4). This outcome makes sense because it requires a longer time for larger oligomers to be formed in sufficient quantities and be detected owing to the aggregation process. We compared these findings with those obtained under conditions where the same seeds were fragmented by sonication. The volume of the oligomers detected by a set of nanopipettes with diameters between 6 and 40 nm ranged between 30 and 6000 nm^3^ corresponding to oligomers smaller than 20 nm. The findings reported in Figure 4 show that fragmented seeds induced the formation of smaller oligomers. However, oligomers with volumes greater than 1000 nm^3^ were detected within the first few minutes of incubation, whereas those with volumes smaller than 200 nm^3^ were detected later. This implies that fragmented seeds are more efficient in producing larger oligomers (>10 nm) than smaller oligomers (<10 nm). A similar trend was previously reported for α‐synuclein, in which the effect of seed structure was also observed.^[^ ^31^ ^]^ To investigate whether fiber maturity influences oligomers formed during the lag phase, similar experiments were carried out by adding seeds harvested from the plateau phase (incubated for 120 h) (Figure 4). When unfragmented seeds were added, the volume aggregate distribution ranged from 30 to 10 000 nm^3^ corresponding to oligomer sizes between 3 and 23 nm. Oligomers with volumes greater than 200 nm^3^ were detected during the first minute of the incubation. In the experiments seeded with fragmented fibers harvested in the plateau phase, the oligomers detected after 30 min of incubation were numerous and smaller than 600 nm^2^. Then, a population of few oligomers with volumes greater than 30 000 nm^3^ (corresponding to oligomer sizes of 34 and 48 nm) was detected after 3 h of incubation. By comparing various seeding conditions, we found that adding unfragmented fibers harvested during the plateau phase resulted in a greater variety of oligomers than those produced during the growing phase. Moreover, fiber fragmentation accelerates the aggregation process, as shown by ThT fluorescence and the early appearance of oligomers, but also induces a change in the volume distribution of the oligomers. These results appear to make sense and are consistent with our expectations. However, it is surprising that only small oligomers were detected 4 harvested in the plateau phase, whereas unfragmented seeds generated larger oligomers. This can be interpreted in two ways: First, sonication at 100 W was used to modify the fiber structure. Thus, the aggregates generated on the fiber surface differed according to the template role of the seeds. Second, the size of the seeds (approximately 30 nm) could limit the size of the oligomers, at least during the time required for their elongation. This could explain the late detection of large oligomers with volumes greater than 30 000 nm^3^.

### 2.3 Analysis of β‐Sheet Structured Aggregates by Confocal Fluorescence Spectroscopy

**Figure 5:**
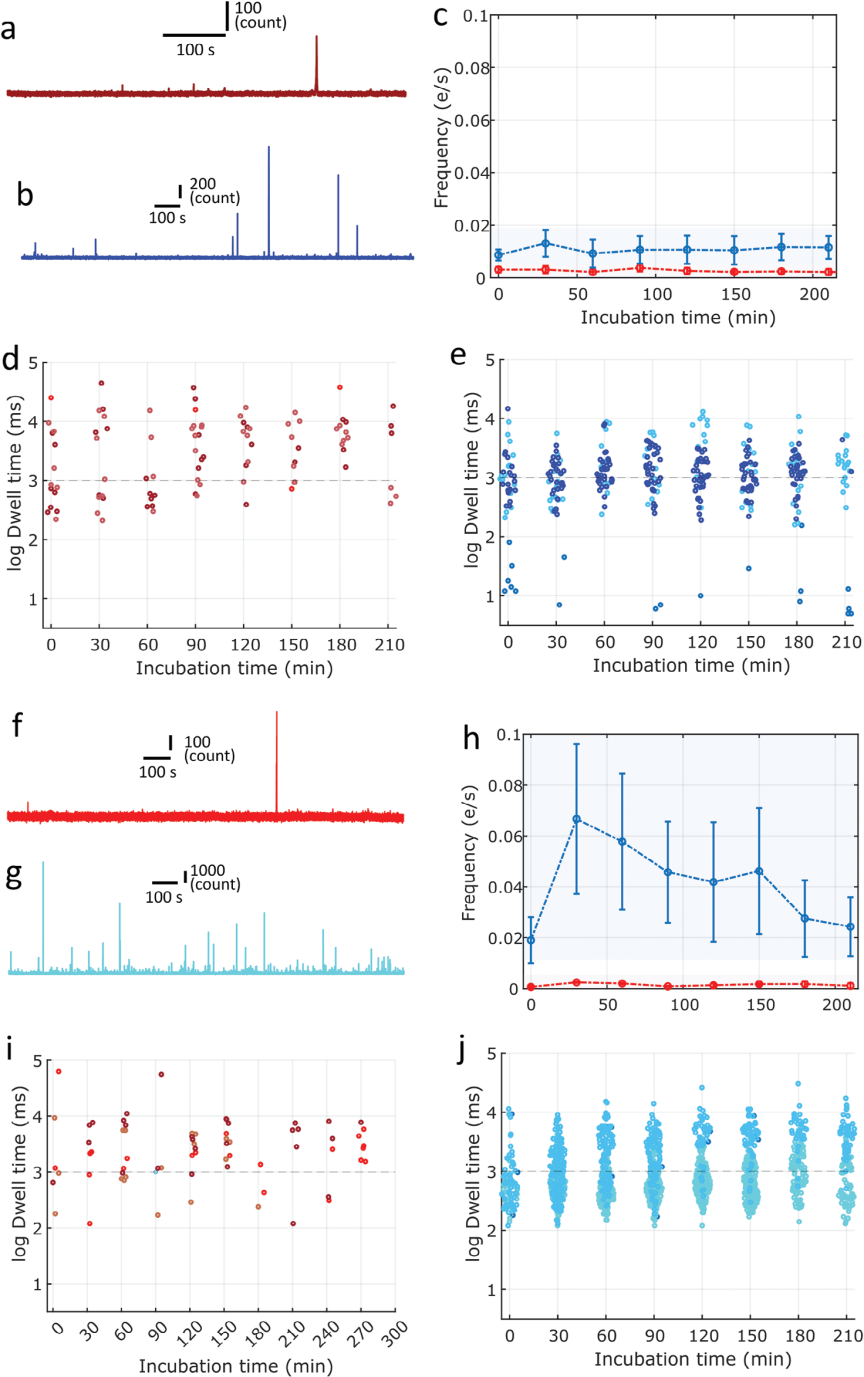
Experimental fluorescence trace for aggregation experiments seeded with 1% seeds harvested at growing phase a) unfragmented and b) fragmented. c) Frequency (number of bursts per second) as a function of incubation time for a triplicate of aggregation experiments seeded with 1% seeds harvested at growing phase unfragmented (blue) and fragmented (red). Dwell time distribution of burst measured for 3 independent experiments seeded with 1% seeds harvested at growing phase d) unfragmented and e) fragmented. Experimental fluorescence trace for aggregation experiments seeded with 1% seeds harvest at plateau phase f) unfragmented and g) fragmented. h) Frequency (number of bursts per second) as a function of incubation time for a triplicate of aggregation experiments seeded with 1% seeds harvested at plateau phase unfragmented (blue) and fragmented (red). Dwell time distribution of burst measured for 3 independent experiments seeded with 1% seeds harvested at plateau phase i) unfragmented and j) fragmented.

As mentioned previously, nanopores allow for the characterization of all oligomers in a range of sizes, regardless of their structure. Nevertheless, to elucidate the secondary nucleation mechanism, it is necessary to obtain specific information on on‐ and off‐pathway oligomers and their evolution during the lag phase.^[^ ^37^ ^]^ This involves specifically investigating oligomers rich in the β‐sheet structure when the ThT fluorescence is too low in bulk to produce a positive signal. To this end, we performed aggregation experiments under strictly the same conditions as the microplate and nanopipette, and detected the aggregates by confocal fluorescence spectroscopy. This single‐molecule technique involves measuring the photon fluorescence burst emitted as soon as an aggregate positive for ThT crosses the confocal volume (**Figure** **5**). These bursts were characterized by their intensity and time. Because the fluorescence intensity depends on the number of ThT molecules, the conformation of the aggregate, and its position in the confocal volume, it is not suitable to use this value to obtain reliable information on the size of the detected object. This justifies the analysis of only the residence time, which is more relevant because it is directly dependent on the friction coefficient of the oligomer when considering the random trajectory of the object in the confocal volume.

For the aggregation experiments with unfragmented seeds (collected in the growing or plateau phases), we observed a low event frequency of < 0.005 events s^−1^ (Figure 5). Intriguingly, the frequencies did not increase with the incubation time, as expected, assuming the formation of an increasing number of amyloid‐competent oligomers. The time distribution of fluorescence burst is broad, ranging from 0.1 to 10 s. Although the number of events detected on triplicates is relatively low, we can note a shift in the time distribution of bursts toward longer values during the first 90 min of incubation. For the experiments carried out with addition of fragmented seeds, the frequency of events is higher, reaching values of about 0.01 and >0.02 events s^−1^ for seeds harvested in the growing phase and plateau phase respectively. At this stage, it is not possible to state that the maturity of the fibers is the cause of this frequency difference because the mature fibers were sonicated at a higher power, producing smaller and more numerous oligomers. We also observed that burst frequency did not increase with incubation time. For the experiments involving unfragmented seeds, the time distributions of the fluorescence bursts were broad, ranging from 0.1 to 10 s, and shifted toward higher values with incubation time. To determine the correlation between the burst duration and size of the detected aggregates, we calibrated the device using a set of fluorescent latex beads with different diameters. The results show that a time interval between 0.1 and 1 s is characteristic of nanoparticles with diameters ranging between 100 and 200 nm, whereas a time interval between 1 and 10 s was obtained for nanoparticles with diameters from 350 to 500 nm (Figure S2, Supporting Information). As oligomers can be composed of independent fibers, a series of calibration experiments were carried out with standard amyloid fibrils with lengths equal to 150 and 250 nm, for which production and characterization have already been reported.^[^ ^28^ ^]^ For both samples, burst duration values were measured around 0.1 s (Figure S2, Supporting Information).

If we interpret the results obtained by confocal fluorescence spectroscopy considering the calibration results, we can state that the detected oligomers have friction coefficients on the order of nanoparticle sizes from 100 to 500 nm. In addition, the dwell times were significantly longer than those obtained for isolated fibers ranging from 100 to 250 nm. This finding is not compatible with the isolated fibers or small oligomers detected by nanopores; thus, we have to consider that the fluorescence bursts are due to the fiber structure in 3D clusters. The fact that the frequency of bursts did not increase with incubation time supports this interpretation, indicating that the source of these fluorescence bursts was limited to added seeds. This interpretation is also supported by the longer fluorescent burst times measured during the first 20 min of incubation with unfragmented seeds, which formed clusters, in contrast to fragmented seeds, as shown by TEM.

### 2.4 Interaction Between Monomers and β‐sheet Structured Aggregates by Molecular Dynamic Simulation

**Figure 6:**
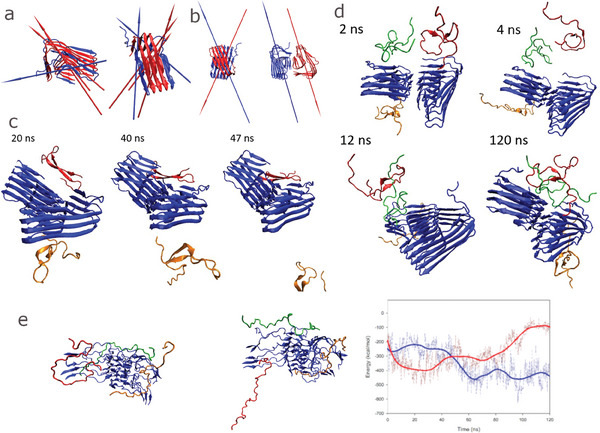
a) Dipolar moments of monomers constituting a substructure of 5OQV: “inside monomers” dipoles being nearly parallel while “edge monomers” show less organized behavior. b) Dipolar moments of both substructures, not entirely antiparallel but on the same plane. All those moments were taken as snapshot but behave identically for the duration of simulations. c) Visualizations of the MD simulations with 2 monomers (red and orange) in the vicinity of a fibril (blue). Only proteins are shown for visibility. The orange monomer is departing the fibril after being attracted to it for 45 ns, it finally adsorbs again on a neighbor periodic fibril 15 ns later after crossing periodic boundaries. d) Visualizations of the MD simulation with 3 monomers (red, orange, and green) in the vicinity of a fibril (blue). Only proteins are shown for visibility. The red monomer starts leaving the fibril before being adsorbed on the fibril, but it interacts with the green monomer to form an disordered dimer. This dimer stays adsorbed, with both monomers interacting with the fibril surface monomer A is red, monomer B is orange, and monomer C is green (the value of pair interactions are reported in Table S1, Supporting Information). e) Visualizations of the 2NAO MD simulations with a monomer partially detaching itself from the fibril and interaction energies between the attached (blue curve) and detached (red curve) monomer and the 2NAO prefibril.

To further understand the interaction between the Aβ42 monomer and β‐sheet‐structured aggregates (considered as seeds, PDB‐ID: 5OQV and 2NAO), 18 (9 for each structure) independent all‐atom molecular dynamics simulations were performed, in which 36 Aβ42 monomers were investigated. For an individual simulation time of 100–160 ns, the monomers did not spontaneously adopt a β‐sheet structure when interacting with the extremities or on the seed side, explaining the long experimental time required to form such amyloid. Nevertheless, their conformations changed to interact with different parts of the aggregate during the simulation. The pair interaction energies reported between the monomers and the seed reached a wide range of different plateaus of stability in the simulation. For the 9 simulations dealing with the 5OQV prefibril structure, the various values from −50 to −650 kcal mol^−1^ obtained for the observed plateau reveal that the conformation of the monomers remains out of equilibrium over the simulation time, which does not seem to be attainable at a reasonable cost. The root‐mean‐square deviation (RMSD) of the monomer stabilized at ≈10 Å for every simulation, regardless of the interaction with the seed. Similar values were obtained for the monomer that relaxed after extraction from the seed. This value seems to be the average deformation reached from any conformation, despite the large range of values observed for the pair interactions. In addition to the RMSD and energy analyses, two interesting cases were observed among the 9 simulated cases. One Aβ42 monomer completely left the seed after being slightly attracted to it (Video S1, Supporting Information). The monomer wanders for 15 ns, from 45 to 60 ns of the simulation, where the pair interaction energies with the seed are 0 ± 20 and 0 ± 1 kcal mol^−1^ for the electrostatic and van der Waals forces, respectively. Then, the Aβ42 monomer crosses the periodic boundaries, is attracted to the seed, and ends up staying there (**Figure** **6**). In a different simulation, an Aβ42 monomer started to leave the seed (Figure 6) and directly interacted with another Aβ42 monomer to form a dimer on the seed side (Video S2, Supporting Information). For the remaining nine simulations involving 2NAO, three distinct monomer configurations were obtained following relaxation and were positioned within the fibril through docking simulations. During these simulations, no monomer was completely detached from the fibril over the entire simulation duration, reaching a maximum of 140 ns. However, multiple monomers underwent partial or significant detachment. Among the 18 simulated monomers, five achieved a plateau of low pair interaction energies (below −150 kcal mol^−1^) after becoming more securely attached to the fibril (as shown in Figure 6 and detailed in Table S2B, Supporting Information). Notably, the monomers that extended their reach (as depicted in Figure 6) were capable of forming interactions and attracting roaming monomers to the fibril and themselves.

The seed used for the simulation (PDB‐ID: 5OQV and 2NAO) consisted of monomers organized in a β‐sheet structure that formed two symmetrical substructures (Figure 6). For 5OQV, the dipole conformation of the two subunits did not change significantly during the 130 ns of the simulation. The dipoles were 310 ± 40 D and 270 ± 50 D for subunits composed of four and five monomers, respectively. Interestingly, the dipoles are coplanar but not antiparallel, as is expected for a stable structure such as an amyloid. This means that several competitions exist between dipolar forces and other chemical forces to maintain the integrity of the structure. With the exception of the edge effect, the substructure displays a high degree of parallelism (Figure 6), which may impede the incorporation of a single monomer into the substructure. This observation suggests that larger pre‐existing structures may be more effective in overcoming dipolar forces than smaller structures in terms of early growth.

### 2.5 Discussion about the Secondary Nucleation Mechanism

**Figure 7:**
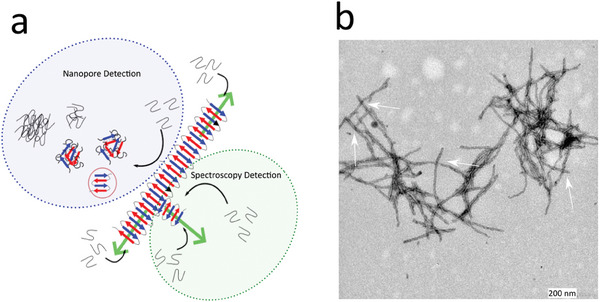
a) Sketch of secondary nucleation that highlights the oligomer species detected by nanopore and confocal fluorescence spectroscopy. b) Transmission electron microscopy of a cluster presented at the start of the exponential phase of aggregation. It is possible to observe a 3D organization of the cluster with the growth of fibers entangled one on top of the other.

In summary, the experiments in the microplate showed a reduction in the aggregation time for samples seeded with preformed aggregates harvested during the growth and plateau phases (Figure 3). Single‐molecule analysis, that is, nanopore and fluorescence, of the oligomers formed during the lag phase provides complementary information. The nanopores revealed the presence of oligomers ranging in size from a few nanometers to 50 nm, with a distribution dependent on the added seeds (Figure 4). Spectroscopic experiments, which are sensitive to oligomers structured in β‐sheets, revealed the presence of large fiber clusters but not small amyloid‐competent oligomers (Figure 5). This implies that, in contrast to larger objects, the oligomers found in the nanopores are not organized in a β‐sheet. Molecular dynamic simulations show that a monomer does not spontaneously adopt a β‐sheet structure when interacting with a seed. Elongation and branching do not seem to be the most favorable mechanisms at the simulation timescale (Figure 6). In addition, a monomer can leave the fiber, making the process dynamic, which is likely to be influenced or sped up by interactions with the environment, notably with other monomers that are likely to be attracted by the fibril. Therefore, we argue that the secondary nucleation mechanism leads to the formation of two types of oligomers on the seed surface (**Figure** **7**). The first population of small oligomers with small β‐sheet structures was released into solution. They are essentially composed of end‐off or non‐pro‐aggregative oligomers, although the presence of end‐on oligomers cannot be completely ruled out. Nanopore analysis showed that the size distribution of these oligomers and their kinetics of formation depended on the properties of the amyloid seeds (maturity, size, and structure). A second population of end‐on oligomers grew directly on the fibers used as seeds. These oligomers, detected by ThT fluorescence, were structured in a β‐sheet, allowing their elongation to form a cluster directly on the parent fiber. This is plausible, because amyloid‐β structures are insoluble in water and form clusters. In contrast, non‐pro‐aggregative oligomers are released in solution because they are more soluble and thus do not gain energy to stay on the parent fiber. These oligomers can dissociate back into monomers rather than maturing into fibrillar species.^[^ ^4^ ^]^ The secondary nucleation mechanism depicted in our experiment, involving the formation of two oligomer populations during the lag phase, confirms the recent one proposed by Thacker et al., based on high‐resolution microscopy analysis.^[^ ^6^ ^]^ Typically, monomers interact with fibril surfaces to form small oligomers that are compatible with parent fibers. The first is quickly released in solution, whereas the second nucleates on the fibril surface and grows into larger aggregates, inducing a large 3D structure. However, it is possible that this process induces fragmentation to create new seeds, explaining why sub‐second bursts of fluorescence are still detected after 1 h of incubation. This interpretation may also explain the nodes between the amyloid fibers observed in TEM (Figure 7), which would not result solely from artifacts due to the sample preparation. In this scheme of the secondary nucleation process, in which the catalytic surface is located on the seed side, it does not appear to be influenced by seed fragmentation, as indicated by the reduction in lag time observed through bulk measurements of ThT fluorescence (Figure 3). As previously mentioned, the formation of β‐sheet structures in amyloids involves elongation, which increases with seed fragmentation. However, in addition to the number of catalytic sites, their accessibility seems to play a crucial role in secondary nucleation. This was evidenced by transmission electron microscopy (TEM) images (Figure 2) and confocal fluorescence spectroscopy, which revealed that unfragmented seeds consist of fiber clusters that exhibit characteristics different from those of individual fibrils after sonication. It is plausible that the low mobility and fiber network of unfragmented seeds make catalytic sites less accessible than mobile individual fibrils. Moreover, crowding in the fiber clusters may hinder the growth of new branches of β‐sheet‐structured fibrils. An alternative hypothesis is that the sites that catalyze the formation of β‐sheets are also involved in the formation of clusters. Regardless of this hypothesis, if β‐sheets grow more rapidly on fragmented seeds than on clusters, the number of catalytic and elongation sites will increase more rapidly over time, accelerating the aggregation process.

## 3 Conclusion

In this study, we aimed to investigate the secondary nucleation mechanism and influence of seed properties on the aggregation of Aβ42 peptides. Four complementary methods are used to achieve this goal. The fluorescence in bulk measurements demonstrated the seeding capacity of seeds and revealed that the aggregation kinetics were modulated by the maturation and fragmentation of the fibers. The nanopipette experiments allowed us to map the volume of the oligomers during the lag phase, showing a strong influence of the seed type. Confocal fluorescence spectroscopy revealed that the size, but not the number, of β‐sheet‐structured aggregates increased with incubation time. Molecular dynamics simulations confirmed that a monomer can leave the fiber and that the organization in the β‐sheet is not spontaneous. These results led us to propose a mechanism of secondary nucleation involving two populations of aggregates: one released in solution and one structuring the β‐sheet growing directly on the fiber. Overall, our study confirms the complexity of the secondary nucleation process, which is a key factor in understanding the general mechanism of amyloid growth. We also demonstrated that the combination of single‐molecule approaches could be an efficient alternative for characterizing a wide range of oligomers. We believe that this methodology can be extended to investigate the impact of promoters and inhibitors of amyloid growth with the possibility of focusing on primary or secondary nucleation. This will provide numerous opportunities for a better understanding of the effects of chemical or physical factors on the aggregation process, testing new drugs, or developing diagnostic tools based on amplification.

## 4 Experimental Section

### Aβ42 Peptide Purification and Aggregation

Monomer purification procedures were performed on Aβ42 peptides (ERI Amyloid Laboratory LLC, Oxford, CT, USA) using a previously described method.^[^ ^32^ ^]^ The Aβ42 peptides were dissolved in a 6.8 M solution of guanidine thiocyanate (Sigma‐Aldrich) to achieve a final concentration of 8.5 mg mL^−1^. The solution was then subjected to sonication at 52 °C for 5 min, followed by dilution with MilliQ water (4 °C) to a concentration of 5 mg mL^−1^ Aβ42 peptides (and 4 M guanidine thiocyanate). The solution was then centrifuged at 10 000 × g (4 °C) for 6 min and the collected supernatant was filtered through a PVDF filter with a diameter of 0.45 µm. The filtrate was then injected into a Superdex 75 Increase 10/300 GL column (GE Healthcare Life Science) equilibrated with 10 mM sodium phosphate buffer (pH 7.4). Monomer purification was performed at a flow rate of 0.5 mL mi^−1^n with FPLC (Cytiva) at 4 °C and detected using UV. The peptide concentration was determined by integrating the area under the curve using the Cytiva software. Monomeric peptides were flash‐frozen in liquid nitrogen and stored at −80 °C in protein LoBind tubes (Eppendorf) until use.

### Preparation and Characterization of Aβ42 Aggregates (Seeds)

The Aβ42 monomer was diluted to 30 µM in a Protein LoBind tube (Eppendorf) to a volume of 600 µL using 10 mM sodium phosphate buffer (pH 7.4). The tubes were then incubated vertically at 25 °C without shaking. Aggregation was monitored using a thioflavin T (ThT) binding assay. Aliquots (20 µL) were withdrawn from the tubes at different aggregation times and mixed with 14 µL 142 mM GlyNaOH buffer (pH 8.3) and 6 µL 100 µM ThT. The fluorescence measurements of the aliquots were performed by placing them in a 96‐well plate of black polystyrene with a clear bottom coated with PEG (Thermo Fisher Scientific) using a Fluoroskan Ascent microplate fluorimeter (Thermo Fisher Scientific). The ThT fluorescence signal of each sample was measured at an excitation wavelength of 445 nm and emission wavelength of 485 nm. The aggregates collected after 73 h and 120 h were aliquoted, flash frozen, and stored at −80 °C until their use in seeding experiments.

### Sonication of Amyloid Aggregates

The samples collected after 73 h of incubation were sonicated for 1 h at room temperature using a classical wet bench sonicator set to 34 W. Similarly, the samples collected after 120 h of incubation were sonicated for 20 min at 100 W using a CupHorn sonicator at 4 °C. To prevent an increase in temperature, a cycle consisting of 7 s of sonication, followed by 3 s of rest, was applied. The samples were then flash‐frozen in liquid nitrogen and stored at −80 °C until use. Transmission electron microscopy was performed on the samples collected before and after sonication for 73 and 120 h, respectively. The samples were deposited onto Formvar carbon‐coated grids, negatively stained with freshly filtered 2% uranyl acetate, and then dried. TEM images were acquired using a JEOL 1400 electron microscope at an accelerating voltage of 80 kV.

### Characterization of the Seeding Capacity of Preformed Aggregates in Microplate

The aggregates collected after 73 h (or 120 h) were diluted in a solution consisting of Aβ42 fresh monomers in NaPh 10 mM buffer. The preformed aggregate and monomer concentrations were 0.04 and 4 µM, respectively. Thioflavin‐T was then added to the solution at a final concentration of 6 µM. The solution was placed in a 96‐well plate with a clear bottom coated with PEG (Thermo Fisher Scientific) at a volume of 100 µL. Fluorescence was monitored using a Fluoroskan Ascent microplate fluorimeter (Thermo Fisher Scientific) with one measurement every 10 min at a temperature of 25 °C without shaking. Three wells were prepared for each condition (triplicate). The aggregation kinetics of the triplicates was fitted using the Boltzmann equation (Equation (1)) To determine the half‐life of aggregation (T_50_). The control conditions consisted of a solution containing only peptide monomers (without the addition of preformed aggregates).

### Nanopipette Pulling and Characterization

Quartz capillaries (OD: 1 mm, ID: 0.3 mm, Sutter Instruments) were fabricated using a laser pipette puller (P‐2000, Sutter Instrument) following two distinct protocols (Table S1 and Figure S1, Supporting Information). The first protocol, which yielded a tip diameter of 14 ± 4 nm, used the 2 lines protocol: HEAT = 750, FIL = 0, VEL = 25, DEL = 128, PUL = 50 and HEAT = 750, FIL = 0, VEL = 10, DEL = 128, PUL = 195; the second protocol, which produced a tip diameter of 48 ±15 nm, used the following parameters: HEAT = 750, FIL = 0, VEL = 25, DEL = 128, PUL = 50, and HEAT = 750; FIL = 0, VEL = 10, DEL = 128, and PUL = 130. It is important to note that the diameter of the capillaries is influenced by various factors, such as room temperature, humidity, and laser alignment of the P‐2000 puller. The tip diameter (r_p_) was estimated using Equation (4):

(4) $$r_{p}=\frac{G}{\kappa \pi \tan\left(\alpha \right)+0.25}\;$$

where *r_p_* is the pipette nanopore, κ is the conductivity of the solution measured using a conductometer (HANNA), and tan(α) is the tangent of the cone angle. The latter was measured after each experiment by epifluorescence microscopy (DM6000 Leica, objective 100x Olympus) after filling the pipette with Rhodamine B. After the pulling process, the pipettes were filled with pure degassed water, according to the filling principle described by Sun et al.^[^ ^38^ ^]^ After complete filling, the conductance of the nanopipettes was measured, and they were coated with saturated L‐DOPA solution (8.5 mg mL^−1^) for 2 h. The nanopipettes were then carefully washed several times with degassed water to remove excess L‐DOPA and characterized with NaCl 1 M and PBS solutions to confirm the success of the functionalization process.

### Seed Amplification Analyses by Nanopipette

Aβ42 monomers were diluted to 4 µM in 1 M NaCl solution or PBS (pH 7.4) with or without seeds (40 nM). The experimental conditions were identical to those used for the microplate experiments. Next, 40 µL of the solution was added to the nanopipette and connected to the working electrode of an EPC10 amplifier (HEKA, Lambrecht, Germany) and probe selector (HEKA, Lambrecht, Germany). The ground electrode was placed in an external reservoir containing 1 M NaCl or PBS (pH 7.4). The current as a function of time was recorded for 10 min under an applied voltage of −500 mV at a sampling rate of 200 kHz and filtered with a Bessel filter at 10 kHz. Then, a 20‐min break was taken without an applied voltage to allow aggregation to occur. This cycle was repeated for the duration of the experiment. The current traces recorded at different incubation times were analyzed using the custom‐made LabVIEW software (Peak Nano Tool). The signal was filtered with a Butterworth filter of 20 kHz order 1, and the baseline fluctuations were corrected with a Savitzky–Golay (order 1) filter to determine the detection threshold, which was set at 4 σ (σ represents the standard deviation of the baseline signal). These events were characterized by their relative blockade amplitudes (ΔI/I) and times (Δt). Statistical analyses were performed using MATLAB script (matlab2022a).

### Seed Amplification Analyses by Confocal Fluorescence Spectroscopy

Aβ42 monomeric units were diluted to 4 µM in a sodium chloride solution containing 1 M NaCl and PBS (pH 7.4), with or without seeding agents (40 nM) and 6.5 µM thymidine (ThT). The experimental conditions were identical to those used for the microplate and nanopipette assays. The fluorescence was recorded as a function of time using a laboratory‐made confocal fluorescence setup. The excitation was provided by a collimated laser diode module (Thorlabs, model CPS450) with a wavelength of 450 nm, which was focused using a UPlanApo 60x/1.20 objective (Olympus). The emitted fluorescence was separated using a 488 nm laser BrightLine single‐edge laser dichroic beam splitter (Di02‐R488‐25×36, Semrock) and collected using a hybrid detector (HPM‐100‐40, Becker and Hickl) connected to a field‐programmable gate array (FPGA) card (EM acquisition card, National Instruments PCI‐7830R R Series Multifunction RIO). The emitted photons were binned with a time scale of 10 ms, and the photon trace was analyzed using the custom‐made LabVIEW software. To detect fluorescence bursts, a threshold of 4 σ (σ is the standard deviation of the baseline signal) was used. The fluorescence burst was characterized by its intensity relative to the number of photons on the peak, the time taken at the peak base, and the area, which is the integration of the total photons emitted during the fluorescence burst. Dwell time calibration was performed using a previously described calibration standard of aβ42 in^[^ ^28^ ^]^ and fluorescent microspheres (fluoresbruite YG microsphere, polysciences) with diameter from 0.1 to 1 µm. The diameter of the nanospheres was controlled by dynamic light scattering (DLS).

### Molecular Dynamic Simulation

Numerical simulations were performed using the classical all‐atom molecular dynamics algorithm, NAMD2.12.^[^ ^39^ ^]^ Visualization and analysis were performed using VMD software. The CHARMM36 force‐field optimization parameters^[^ ^40^ ^]^ were employed in all simulations. Langevin dynamics and piston methods were used to maintain the system temperature and pressure at 300 K (Langevin dynamics)^[^ ^41^ ^]^ and 1 atm (Langevin piston),^[^ ^42^ ^]^ respectively. Long‐range electrostatic forces were calculated using the classical particle mesh Ewald (PME) method with a 1.2 Å grid spacing and fourth‐order spline interpolation. The integration time step is 1 fs. Periodic boundary conditions were applied in three spatial directions for each simulation. The crystal structure of the Aβ42 protofibril, 5OQV,^[^ ^43^ ^]^ and 2NAO^[^ ^44^ ^]^ were used to model the fibers in the simulations. The most recent structure (5OQV) that presented a resolution of 4Å appeared as the best candidate for pre‐fibril study, while determined in acidic conditions with organic solvent. For this structure, the structure according to PDB2PQR tools (PROPKA algorithm)^[^ ^45^ ^]^ and CHARMMGUI procedure^[^ ^46^ ^]^ was rebuilt to obtain a structure more realistic for neutral solvent. For 2NAO, the structure that was used was the common AutoPSF plug‐in to complete the missing segments and add hydrogen fit for an aqueous and saline environment. To generate monomers, a single monomer was isolated from each structure and allowed to relax in water for 10 ns, stabilizing with a root‐mean‐square deviation (RMSD) of 10 Å. The conformations obtained from this structure were then positioned around their respective fibrils at various angles and positions to create three different systems with one, two, or three monomers surrounding the fibril (Figure 6). For each simulation, 3 different conformations were obtained after relaxation and positioned through docking computations performed on the GRAMM web server.^[^ ^47^ ^]^ Each of the 6 system was run three times with different random seeds to observe the various behaviors for 100–160 ns of the simulated time. These systems were placed in a periodic box filled with water of dimensions 105 × 80 × 80 Å^3^ (5OQV) and 125 × 115 × 100 Å^3^ (2NAO), which had been ionized with a K^+^ and Cl^−^ 0.15 M electrolyte solvent. The system contains approximately 77 000 atoms and 150 000 (2NAO) atoms. The completely functionalized system was subsequently optimized using three successive procedures. First, the total energy of the system was minimized at 0 K. Next, the system was gradually heated until it reached a temperature of 300 K. Finally, the system was allowed to evolve in the NPT ensemble and physical observables were determined using time averages.

## Conflict of Interest

The authors declare no conflict of interest.

## Supporting information

Supporting Information

Supplemental Video 1

Supplemental Video 2

## Data Availability

The data that support the findings of this study are available from the corresponding author upon reasonable request.
