# Supplementary material for: Secondary Nucleation of Aβ Revealed by Single‐Molecule and Computational Approaches
Source: Adv Sci (Weinh). 2024 Aug 19;11(39):2404916. doi: 10.1002/advs.202404916 (PMC11497034; doi:10.1002/advs.202404916)
Supplement: Supplementary file 1 — Supporting Information [file ADVS-11-2404916-s003.docx]

Supporting Information

Secondary nucleation of Aβ revealed by single molecule and computational approaches.

Nathan Meyer, Nicolas Arroyo, Lois Roustan, Jean-Marc Janot, Saly Charles-Achille, Joan Torrent, Fabien Picaud, Sébastien Balme*.


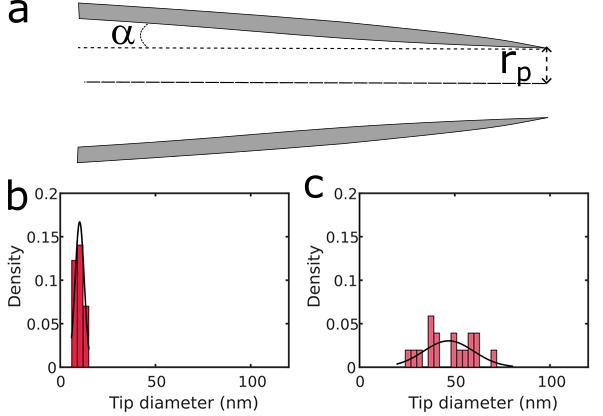


Figure S1: a) Scheme of a nanopipette with the tip diameter (r_p_) estimated using equation 1, α corresponding cone angle. Distribution of pipette diameter pulled following b) protocol #1 (HEAT = 750, FIL = 0, VEL = 25, DEL = 128, and PUL = 50 and HEAT = 750, FIL = 0 VEL = 10, DEL = 128, and PUL = 195) and c) protocol #2 (HEAT = 750; FIL = 0, VEL = 25, DEL = 128, and PUL = 50 and HEAT = 750; FIL = 0, VEL = 10, DEL = 128, and PUL = 130). The angle α was estimated from optical microscopy images according to the ref ^[1]^. The mean values of α 4° and 5.4° are found for the protocols #1 and #2 Protocol #2 respectively. We notice that for each nanopipette the angle was measured after the experiment to obtain the most precise value of the *r_p_*.

| Protocol | Diameter (nm) | Experience |
| --- | --- | --- |
| #2 | 28 | Seeding incubated 73h unfragmented |
| #1 | 7 | Seeding incubated 73h unfragmented |
| #1 | 6 | Seeding incubated 73h unfragmented |
| #2 | 61.21 | Seeding incubated 73h unfragmented |
| #2 | 33 | Seeding incubated 73h unfragmented |
| #1 | 7 | Seeding incubated 73h fragmented |
| #2 | 20 | Seeding incubated 73h fragmented |
| #1 | 15 | Seeding incubated 73h fragmented |
| #2 | 31 | Seeding incubated 73h fragmented |
| #2 | 25 | Seeding incubated 73h fragmented |
| #1 | 15 | Seeding incubated 120h unfragmented |
| #1 | 4 | Seeding incubated 120h unfragmented |
| #1 | 4 | Seeding incubated 120h unfragmented |
| #2 | 37 | Seeding incubated 120h unfragmented |
| #1 | 6 | Seeding incubated 120h fragmented |
| #1 | 7 | Seeding incubated 120h fragmented |
| #1 | 10 | Seeding incubated 120h fragmented |
| #2 | 57 | Seeding incubated 120h fragmented |
| #2 | 44 | Control |
| #2 | 40 | Control |
| #1 | 6 | Control |
| #1 | 4 | Control |
| #1 | 10 | Control |

Table S1: List of nanopipette used for the experiments


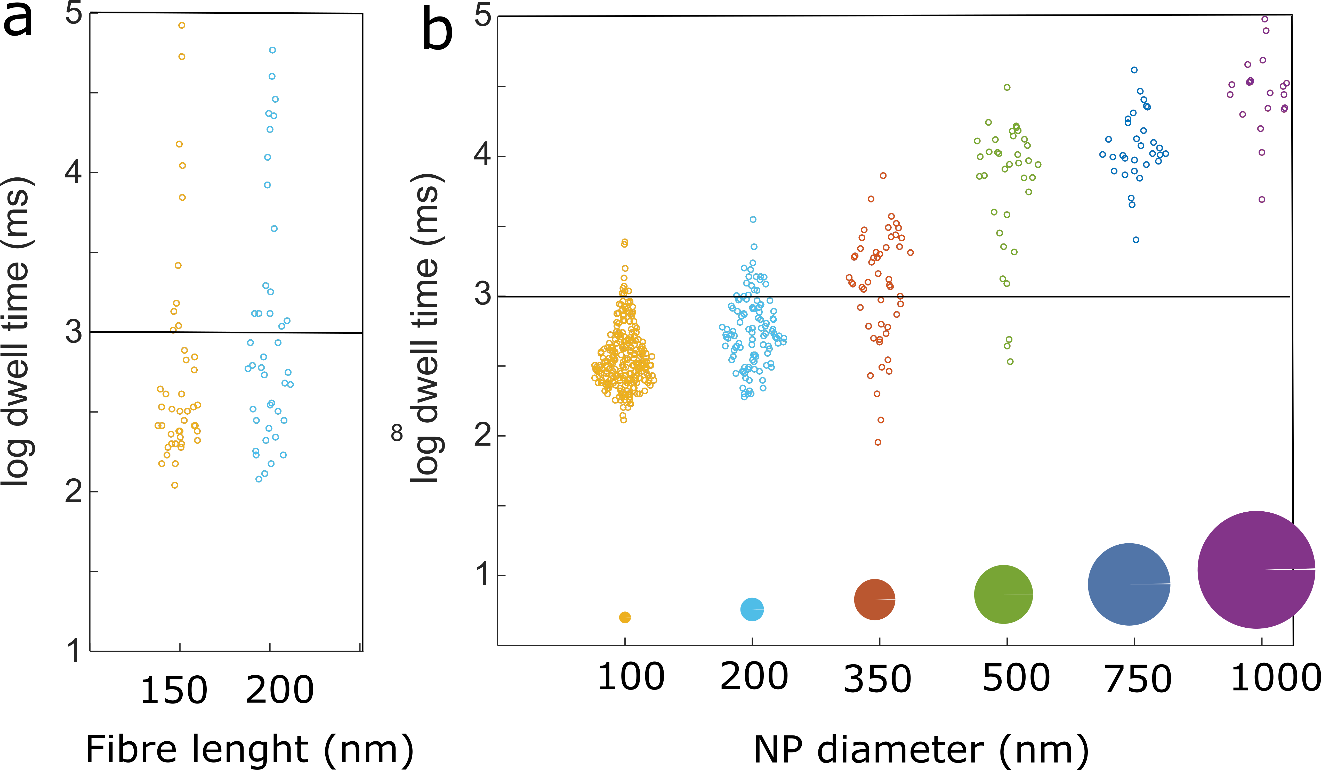


Figure S2: dwell times distribution of a) Aβ42 fiber with length about 150 nm and 200 nm. The description of experimental protocole to obtained these calibrated Aβ42 fiber and their characterization by TEM including the size distribution of was previously reported (see ref ^[2]^). b) spherical calibrated nanoparticle (fluoresbrite YG) in the confocal volume as function of their diameter. The nanoparticle diameter was controlled using dynamic light scattering (DLS).

Table A (5OQV)

Table B (2NAO)

Table S2: Pair interaction energies (van der Waals (vdW) and electrostatic interactions, in kcal/mol) of every monomer with the 5OQV (A) and 2NAO (B) fibrils. As simulations are out of equilibrium, only plateaux of stabilized energies are proposed with their respective duration. When 2 different plateaux are visible, the one on the left is the first one attained by the monomer. Using Fig 6d and 6e colours: monomer A is red, monomer B is orange and monomer C is green. In simulations 7, 8 and 9 of Table A (5OQV), monomers A and C are close enough to have interactions between themselves and as such values for monomer A must not be directly compared with simulations 1 to 6.

**References**

[1] S. Charles-Achille, J.-M. Janot, B. Cayrol, S. Balme, *Chembiochem a European journal of chemical biology* **2024**, *25*, e202300748.

[2] I. Abrao-Nemeir, N. Meyer, A. Nouvel, S. Charles-Achille, J.-M. Janot, J. Torrent, S. Balme, *Biophysical Chemistry* **2023**, *300*, 107076.
